# Sustained natural immunity following SARS-CoV-2 infection against severe COVID-19 outcomes and symptomatic reinfection: analyses of national data for Brazil and Scotland

**DOI:** 10.1136/bmjopen-2025-104057

**Published:** 2025-07-16

**Authors:** Fasih Haider, Thiago Cerqueira-Silva, Kirsten J Hainey, Tristan Millington, Syed Ahmar Shah, Vincius de Araujo Oliveira, Neil Pearce, Mauricio L Barreto, Viviane Sampaio Boaventura, Srinivasa Vittal Katikireddi, Chris Robertson, Manoel Barral-Netto, Aziz Sheikh

**Affiliations:** 1 Usher Institute, the University of Edinburgh, Edinburgh, UK; 2 Laboratório de Medicina e Saúde Pública de Precisão, Fundação Oswaldo Cruz, Salvador, Brazil; 3 London School of Hygiene & Tropical Medicine, London, UK; 4 MRC/CSO Social and Public Health Sciences Unit, University of Glasgow, Glasgow, UK; 5 Public Health Scotland, Glasgow, UK; 6 Center of Data and Knowledge Integration for Health (Cidacs), Fiocruz, Salvador, Brazil; 7 Faculty of Medicine, Federal University of Bahia, Salvador, Brazil; 8 Medical Statistics, London School of Hygiene & Tropical Medicine, London, UK; 9 Federal University of Bahia, Salvador, Brazil; 10 Department of Mathematics and Statistics, University of Strathclyde, Glasgow, UK; 11 Nuffield Department of Primary Care Health Sciences, University of Oxford, Oxford, UK

**Keywords:** COVID-19, PUBLIC HEALTH, Epidemiology

## Abstract

**Abstract:**

**Objectives:**

SARS-CoV-2 infection provides protection against reinfection and severe COVID-19 disease; however, this protective effect may diminish over time. We assessed waning of natural immunity conferred by previous infection against severe disease and symptomatic reinfection in Brazil and Scotland.

**Design:**

We undertook a test-negative design study and nested case–control analysis to estimate waning of natural immunity against severe COVID-19 outcomes and symptomatic reinfection using national linked datasets. We used logistic regression to estimate ORs with 95% CIs. A stratified analysis assessed immunity during the Omicron dominant period in Brazil.

**Setting and participants:**

We included data from the adult populations of Brazil and Scotland from 1 June 2020 to 30 April 2022.

**Outcome measures:**

Severe COVID-19 was defined as hospitalisation or death. Reinfection was defined as reverse-transcriptase PCR or rapid antigen test confirmed at least 120 days after primary infection.

**Results:**

From Brazil, we included 30 881 873 tests and 1 301 665 severe COVID-19 outcomes, and from Scotland, we included 1 520 201 tests and 7988 severe COVID-19 outcomes. Against severe outcomes, sustained protection was observed for at least 12 months after primary SARS-CoV-2 infection with little evidence of waning: <6 months postprimary infection: Brazil OR 0.10 (95% CI 0.09 to 0.11), Scotland OR 0.01 (95% CI 0.00 to 0.05); >12 months postprimary infection: Brazil OR 0.12 (95% CI 0.10 to 0.14), Scotland OR 0.03 (95% CI 0.02 to 0.04). For symptomatic reinfection, Brazilian data demonstrated evidence of waning in the 12 months following primary infection, although some residual protection remained beyond 12 months: <6 months postprimary infection: OR 0.19 (95% CI 0.19 to 0.20); >12 months postprimary infection: OR 0.42 (95% CI 0.40 to 0.43). The greatest reduction in risk of SARS-CoV-2 infection was in individuals with hybrid immunity (history of previous infection and vaccination), with sustained protection against severe outcomes at 12 months postprimary infection. During the Omicron dominant period in Brazil, odds of symptomatic reinfection were higher and increased more quickly over time when compared with the overall study period, although protection against severe outcomes was sustained at 12 months postprimary infection (whole study: OR 0.12 (95% CI 0.10 to 0.14); Omicron phase: OR 0.15 (95% CI 0.12 to 0.19)).

**Conclusion:**

Cross-national analyses demonstrate sustained protection against severe COVID-19 disease for at least 12 months following natural SARS-CoV-2 infection, with vaccination further enhancing protection. Protection against symptomatic reinfection was lower with evidence of waning, but there remained a protective effect beyond 12 months from primary infection.

**STRENGTHS AND LIMITATIONS OF THIS STUDY**

- This study uses linked population datasets, which provide a comprehensive dataset with good population coverage and completeness, across the Brazilian and Scottish populations.
- Conducting harmonised analyses in two different international settings, with differing vaccine schedules and patterns of dominant circulating viral variants, provides assurance in the findings.
- Inadequate documentation of the history of previous SARS-CoV-2 infection may result in misclassification of study participants and affect our effect estimates.

## Background

 Protection against SARS-CoV-2 infection can be acquired through natural infection or vaccination. In vitro studies have demonstrated persistency of neutralising antibodies against SARS-CoV-2 for months after initial infection early in the pandemic.^1^ However, the profile of neutralising antibodies has evolved with the emergence of new variants of concern. Healthcare workers with serological evidence of previous infection have a reduced risk of SARS-CoV-2 infection, with a protective effect sustained for several months after primary infection.^2^ Vaccine effectiveness studies have established that vaccination against SARS-CoV-2 reduces the risk of both severe disease and symptomatic infection.^3^,^6^

Waning of protection from natural infection and vaccination against SARS-CoV-2 increases the risk of reinfection. Using real-world data, vaccine effectiveness studies have demonstrated a waning of protection against both severe COVID-19 disease outcomes (ie, hospitalisation or death) and symptomatic infection within months of vaccination,^7^,^9^ prompting the provision of booster vaccination doses in many countries. The emergence of the Omicron variant in late 2021 marked a change in the profile of vaccine effectiveness against SARS-CoV-2 infection, with lower effectiveness and more rapidly waning vaccine effectiveness compared with the Delta variant.^10^ The waning of natural immunity following previous SARS-CoV-2 infection has been less well characterised, but evidence is emerging to suggest potential waning over time, particularly against symptomatic reinfection.^11^ However, protection against severe disease appears to be well sustained despite waning of immunity against reinfection in studies conducted in the pre-Omicron era.^12 13^ As with vaccine effectiveness, immunity conferred by previous infection appears to offer less protection against reinfection with the Omicron variant, although there is evidence of sustained protection against severe outcomes.^11^

Throughout the COVID-19 pandemic, understanding the duration of protection against SARS-CoV-2 reinfection from both previous natural infection and vaccination was vital in influencing the implementation of preventative measures. These questions remain of ongoing public health importance in determining future vaccination efforts, anticipating future disease burden and informing the deployment of other interventions to minimise harm from the disease.

Quantifying the effects of waning of natural immunity against SARS-CoV-2 infection over time is challenging because of potential confounding from the changing profile of dominant viral variants and changes in the susceptible population with the implementation of vaccination programmes. Performing harmonised analyses across differing international contexts, where the timing of circulating variants and vaccination schedules differed, can help provide greater assurance that observed findings are not due to residual confounding if consistent patterns are observed. We chose to conduct our analyses in Brazil and Scotland based on shared availability of robust population-wide linked datasets, coupled with differing socioeconomic structures and variability in vaccination programmes, circulating subvariants of Omicron, and population characteristics such as age structure, testing policy and vaccination status. We, therefore, aimed to investigate the waning of natural immunity against severe COVID-19 disease following previous natural SARS-CoV-2 infection using national datasets from two countries, Brazil and Scotland.

## Methods

This study is reported following the Strengthening the Reporting of Observational Studies in Epidemiology guideline (online supplemental table S1).^14^ Our primary analytical approach applied a test-negative design (TND) case–control study in national administrative datasets for Brazil and Scotland.

### Study databases

For the Brazilian analysis, data from three deterministically linked national administrative databases were used. The COVID-19 Vaccination Campaign (SI-PNI) and Acute Respiratory Infection Suspected Cases (e-SUS-Notifica) provided clinical and laboratory data on suspected and confirmed COVID-19 cases, and Severe Acute Respiratory Infection/Illness (SIVEP-Gripe) provided data on COVID-19 hospital admissions and deaths.

For the Scottish analysis, we used data from the Early Pandemic Evaluation and Enhanced Surveillance of COVID-19 (EAVE II) platform.^15^ This combined national linked health datasets, including data from general practice, vaccination records, hospital admissions and laboratory testing results. EAVE II includes data from 5.4 million people in Scotland linked through a single unique identifier, which represented population coverage of around 99%. Testing data from both countries included results for SARS-CoV-2 reverse-transcriptase PCR (RT-PCR) and rapid antigen tests. However, for the Scottish analysis, only positive rapid antigen tests were included due to incomplete recording of negative rapid antigen test results.

### Study design and variables

In our (TND) case–control study, cases were defined as symptomatic individuals with a valid positive SARS-CoV-2 test result, and controls were symptomatic individuals with a negative SARS-CoV-2 RT-PCR test result. All adults aged 18 years or older, who reported symptoms of acute respiratory illness and underwent RT-PCR or rapid antigen tests for SARS-CoV-2 between 1 June 2020 and 30 April 2022 were considered eligible for inclusion in the TND analysis. Reinfection with SARS-CoV-2 was defined as a positive RT-PCR or rapid antigen test more than 120 days after an individual’s initial positive SARS-CoV-2 test, mirroring the time period used to define reinfection by the Office for National Statistics in the UK.^16^ Therefore, repeated positive tests within 120 days of an initial positive result were excluded. In addition, there were other exclusion criteria applied to both individuals and tests (data flow diagram in online supplemental figure S1). Individuals were excluded if they had missing data for essential covariates or were aged under 18 years. Tests were excluded if the individual had received four doses of a COVID-19 vaccination; the test was taken within 13 days of receiving a first vaccination dose; where a negative test occurred within 14 days of another negative test; and where a negative test was followed by a positive test within 7 days.

In addition to our primary analysis, a nested case–control (NCC) design was used to investigate time from primary infection to severe COVID-19 outcomes. In each country, a cohort of adults aged ≥18 years with evidence of a previous positive SARS-CoV-2 test was established. Cases were defined as individuals with evidence of severe COVID-19 outcomes following reinfection with SARS-CoV-2 and were matched with controls from the cohort using incidence-density sampling based on age, sex, geographical location (either General Practitioner (GP) practice or municipality) and date of hospital admission of the case. The exclusion criteria were missing data for essential covariates, individuals with more than 10 RT-PCR tests in a 3-month period (used as a proxy for health and social care workers participating in occupational screening and facing differential risks of exposure), inconsistent vaccination records and, in Brazil, individuals identified as having the Omicron variant as index infection because of insufficient follow-up time in this group (<120 days) (data flow diagram in online supplemental figure S2).

### Outcomes

For the TND analysis, our primary outcome was symptomatic COVID-19 infection. Our secondary outcome was severe COVID-19 disease (hospitalisation or death). COVID-19 hospitalisation was defined as an admission within 14 days of a positive RT-PCR test or with a COVID-19-related International Classification of Diseases (ICD-10) code (online supplemental table S2), and COVID-19 deaths were defined as deaths within 28 days of a positive RT-PCR or a COVID-19 ICD-10 code recorded on the death certificate.

In the NCC analysis, the primary outcome was severe COVID-19 disease.

### Exposures

Our primary exposure was time since previous SARS-CoV-2 infection (<6 months, 6–11 months and ≥12 months). Exposure status was also classified according to the number of doses of SARS-CoV-2 vaccine received (one, two or three), although vaccine type was not considered.

### Statistical analysis and reporting

For the TND analysis, logistic regression models were used to calculate ORs for odds of severe outcomes and infection, and their associated 95% CI, adjusted for age (in 5-year intervals), sex, calendar week, number of QCOVID risk groups (Scotland)^17^ or number of medical comorbidities (Brazil) and geographical region. To investigate waning of natural immunity against the Omicron variant, a stratified analysis was conducted using data from 1 January 2022 to 30 April 2022 when the Omicron variant was the dominant circulating strain in Brazil. We did not have enough data to stratify by variant in Scotland; however, Omicron was also the dominant variant of concern during this time period.^18^

In the NCC analysis, conditional logistic regression models were used to calculate OR and their 95% CI comparing odds of severe outcomes between cases and controls, adjusted for number of comorbidities and number of COVID-19 vaccine doses received (0–3). Additionally, interaction analysis was undertaken to investigate the role of time elapsed since first infection and variant type.

All analyses were performed using R statistical software (V.4.1.1). All data were anonymised, and analyses were conducted within secure analytical environments. The statistical analysis plan was agreed prior to commencement of analysis, and all statistical code is available on the EAVE II GitHub page (https://github.com/EAVE-II).

### Patient and public involvement

Our findings have been shared with the EAVE II patient and public involvement volunteers for comment and feedback.

## Results

The characteristics of the study population for the primary TND analysis are described in table 1 (online supplemental table S3). There were 30 881 873 tests in Brazil and 1 520 201 tests in Scotland included. During the study period, there were 1 309 653 severe COVID-19 outcomes: these comprised of 1 253 772 hospitalisations and 363 648 deaths in Brazil and 7743 hospitalisations and 882 deaths in Scotland (data flow diagram in online supplemental figure S1).

**Table 1:** Characteristics of the study population for the test-negative design analysis for Brazil and Scotland.

Characteristic	Levels	Brazil		Scotland	
		Positive tests	Negative tests	Positive tests	Negative tests
Total		14 264 991	16 616 882	1 069 886	450 315
Sex	Female	7 716 781 (54.1%)	9 570 294 (57.6%)	594 293 (55.5%)	285 001 (63.3%)
Age (years)	18–59	11 967 450 (83.9%)	14 395 855 (86.6%)	905 444 (84.6%)	414 759 (92.1%)
	59+	2 297 541 (16.1%)	2 221 027 (13.4%)	164 442 (15.4%)	35 556 (7.9%)
Test type	RT-PCR	7 011 819 (49.2%)	8 178 322 (49.2%)	748 434 (70.0%)	450 315 (100.0%)
	Rapid antigen test	7 253 172 (50.8%)	8 438 560 (50.8%)	321 452 (30.0%)	n/a
Number of comorbidities	0	12 234 575 (85.8%)	14 445 819 (86.9%)	721 363 (67.4%)	279 821 (62.1%)
	1	1 438 900 (10.1%)	1 654 113 (10.0%)	281 712 (26.3%)	133 720 (29.7%)
	2	474 965 (3.3%)	418 894 (2.5%)	57 463 (5.4%)	31 584 (7.0%)
	3+	116 551 (0.8%)	98 056 (0.6%)	9348 (0.9%)	5190 (1.2%)
Diabetes		694 833 (4.9%)	614 726 (3.7%)	41 552 (3.9%)	14 105 (3.1%)
Obesity		265 652 (1.9%)	211 093 (1.3%)	149 794 (14.0%)	70 519 (15.6%)
Immunosuppression		105 451 (0.7%)	141 196 (0.8%)	28 616 (2.7%)	12 102 (2.7%)
Chronic respiratory disease		375 372 (2.6%)	612 316 (3.7%)	55 (0.0%)	44 (0.0%)
Cardiac disease		1 234 453 (8.7%)	1 150 246 (6.9%)	0	0
Chronic kidney disease		80 658 (0.6%)	74 033 (0.4%)	12 916 (1.2%)	3061 (0.7%)
Hospitalisation		1 253 772 (8.8%)	369 171 (2.2%)	7734 (0.7%)	64 (0.0%)
Death		363 648 (2.5%)	93 271 (0.6%)	882 (0.1%)	*
Severe outcome		1 301 665 (9.1%)	387 021 (2.3%)	7998 (0.7%)	64 (0.0%)

* Suppressed on account of small event numbers.

RT-PCR, reverse-transcriptase PCR.

### Risk of SARS-CoV-2 symptomatic reinfection

Compared with those without a history of SARS-CoV-2 infection or vaccination, previous natural infection with SARS-CoV-2 reduced the odds of both severe COVID-19 and symptomatic disease in Scotland and Brazil <6 months postprimary infection, demonstrating the protection conferred by natural immunity (severe disease: Brazil OR 0.10 (95% CI 0.09 to 0.11), Scotland OR 0.01 (95% CI 0.00 to 0.05); symptomatic infection: Brazil OR 0.19 (95% CI 0.19 to 0.20), Scotland OR 0.09 (95% CI 0.08 to 0.09)) (table 2). Previous natural infection showed a greater reduction in odds of severe outcomes compared with symptomatic reinfection.

**Table 2:** Test-negative design analysis demonstrating OR and associated 95% CIs for risk of symptomatic infection and severe disease outcomes by history of previous infection and vaccination status in Brazil and Scotland.

	Symptomatic COVID-19			Severe COVID-19		
	OR	LCL	UCL	OR	LCL	UCL
Brazil						
Unvaccinated and no natural infection (reference)	1.00	–	–	1.00	–	–
Only natural infection <6 months previously	0.19	0.19	0.20	0.10	0.09	0.11
Only natural infection 6–11 months previously	0.23	0.22	0.23	0.08	0.08	0.09
Only natural infection ≥12 months previously	0.42	0.40	0.43	0.12	0.10	0.14
1 vaccination only	0.69	0.69	0.70	0.36	0.36	0.36
1 vaccination+previous infection <6 months previously	0.14	0.13	0.14	0.03	0.03	0.03
1 vaccination+previous infection 6–11 months previously	0.22	0.22	0.22	0.03	0.03	0.04
1 vaccination+previous infection ≥12 months previously	0.33	0.32	0.34	0.05	0.05	0.06
2 vaccinations only	0.62	0.62	0.63	0.23	0.22	0.23
2 vaccinations+previous infection <6 months previously	0.21	0.21	0.22	0.03	0.02	0.03
2 vaccinations+previous infection 6–11 months previously	0.31	0.31	0.32	0.03	0.02	0.03
2 vaccinations+previous infection ≥12 months previously	0.41	0.41	0.42	0.04	0.04	0.05
3 vaccinations only	0.45	0.44	0.45	0.14	0.14	0.14
3 vaccinations+previous infection <6 months previously	0.14	0.13	0.14	0.02	0.02	0.03
3 vaccinations+previous infection >6 months previously	0.24	0.24	0.25	0.03	0.02	0.03
Scotland						
Unvaccinated and no natural infection (reference)	1.00	–	–	1.00	–	–
Only natural infection <6 months previously	0.09	0.08	0.09	0.01	0.00	0.05
Only natural infection 6–11 months previously	0.08	0.07	0.08	0.02	0.01	0.03
Only natural infection ≥12 months previously	0.04	0.03	0.04	0.03	0.02	0.04
1 vaccination only	0.59	0.58	0.60	0.19	0.17	0.22
1 vaccination+previous infection <6 months previously	0.03	0.03	0.04	0.01	0.00	0.06
1 vaccination+previous infection 6–11 months previously	0.05	0.05	0.05	0.01	0.00	0.03
1 vaccination+previous infection ≥12 months previously	0.29	0.25	0.32	*	*	*
2 vaccinations only	0.41	0.40	0.41	0.13	0.12	0.14
2 vaccinations+previous infection <6 months previously	0.03	0.03	0.03	0.00	0.00	0.01
2 vaccinations+previous infection 6–11 months previously	0.11	0.10	0.11	0.01	0.00	0.02
2 vaccinations+previous infection ≥12 months previously	5.00	5.00	5.00	*	*	*
3 vaccinations only	0.23	0.22	0.23	0.07	0.06	0.08
3 vaccinations+previous infection <6 months previously	0.10	0.10	0.11	0.01	0.00	0.04
3 vaccinations+previous infection >6 months previously	5.00	5.00	5.00	0.11	0.05	0.24

* indicates no events observed in the exposed group, suggesting uncertainty due to sparse data.

LCL, lower confidence limit; UCL, upper confidence limit.

### Waning of immunity following primary infection

When examined by duration of protection after primary natural infection, there was evidence of waning over time of natural immunity against symptomatic reinfection in the Brazilian data (symptomatic disease <6 months postprimary infection: OR 0.19 (95% CI 0.19 to 0.20); 6–11 months postprimary infection: OR 0.23 (95% CI 0.22 to 0.23); ≥12 months postprimary infection: OR 0.42 (95% CI 0.40 to 0.43)). However, this pattern of waning was not observed in the Scottish data for symptomatic reinfection (symptomatic disease <6 months postprimary infection: OR 0.09 (95% CI 0.08 to 0.09); 6–11 months postprimary infection: OR 0.08 (95% CI 0.07 to 0.08); ≥12 months postprimary infection: OR 0.04 (95% CI 0.03 to 0.04)). Neither country demonstrated clear evidence of waning against severe outcomes in the subgroups at increasing time intervals following primary infection. Instead, data demonstrated sustained protection against severe outcomes from reinfection beyond 12 months (Brazil: <6 months postprimary infection: OR 0.10 (95% CI 0.09 to 0.11), 6–11 months postprimary infection: OR 0.08 (95% CI 0.08 to 0.09), >12 months postprimary infection: OR 0.12 (95% CI 0.10 to 0.14); Scotland: <6 months postprimary infection: OR 0.01 (95% CI 0.00 to 0.05), 6–11 months postprimary infection: OR 0.02 (95% CI 0.01 to 0.03), >12 months postprimary infection: OR 0.03 (95% CI 0.02 to 0.04)).

### Risk in vaccinated individuals

The lowest odds of severe disease outcomes were observed in the groups with a history of both vaccination and previous SARS-CoV-2 infection. Although the Brazilian data suggested waning of immunity over time against symptomatic reinfection, there was sustained protection against severe disease at least 12 months after primary infection in the vaccinated population (Brazil: one vaccination ≥12 months postprimary infection: OR 0.05 (95% CI 0.05 to 0.06), two vaccinations ≥12 months postprimary infection: OR 0.04 (95% CI 0.04 to 0.05)).

People who experienced both vaccination and natural infection had a greater reduction in odds of symptomatic reinfection than for vaccination or natural infection alone. There was evidence of waning of protection as more time elapsed from primary infection, although a sustained protective effect was demonstrated by reduced odds of symptomatic reinfection at 12 months postprimary infection in the Brazilian data (Brazil: one vaccination +>12 months postprimary infection: OR 0.33 (95% CI 0.32 to 0.34), two vaccinations +>12 months postprimary infection: OR 0.41 (95% CI 0.41 to 0.42)).

### Risk of infection during Omicron variant phase

During the Omicron dominant period, there were 4 946 937 positive tests in the Brazilian dataset and 102 640 severe outcomes with 97 384 hospitalisations and 33 078 deaths (online supplemental table S4). When compared with the whole study period, the proportion of severe outcomes was lower during the Omicron dominant phase (2.1% during Omicron phase vs 9.1% during whole study).

Protection against infection during the Omicron period was lower against symptomatic reinfection than during the whole study period, at all time points and with all combinations of vaccination and natural infection history (table 3). The same patterns of waning and hybrid immunity were observed against Omicron infection as in the whole study period, although the magnitude of effect was less for all exposures. Against severe disease, previous infection conferred protection similar in magnitude to those seen in the whole study period at 12 months postprimary infection (whole study: OR 0.12 (95% CI 0.10 to 0.14); Omicron phase: OR 0.15 (95% CI 0.12 to 0.19)). Restriction to the pre-Omicron phase in the Brazilian data demonstrated differences in pre-Omicron immunity against symptomatic COVID-19, where the odds of infection were lower at all time points in those with evidence of previous infection, regardless of vaccination status, when compared with the analysis of the whole period analysis (online supplemental table S5).

**Table 3:** Test-negative design analysis demonstrating ORs and associated 95% CIs for risk of symptomatic infection and severe disease outcomes by history of previous infection and vaccination status in Brazil during the Omicron dominant phase

	Symptomatic COVID-19			Severe COVID-19		
	OR	LCL	UCL	OR	LCL	UCL
Unvaccinated and no natural infection (reference)	1.00	–	–	1.00	–	–
Only natural infection <6 months previously	0.47	0.43	0.51	0.14	0.08	0.24
Only natural infection 6–11 months previously	0.63	0.61	0.66	0.08	0.06	0.11
Only natural infection ≥12 months previously	0.79	0.76	0.82	0.15	0.12	0.19
1 vaccination only	0.83	0.82	0.83	0.41	0.40	0.43
1 vaccination+previous infection <6 months previously	0.37	0.35	0.39	0.12	0.07	0.18
1 vaccination+previous infection 6–11 months previously	0.49	0.48	0.50	0.07	0.06	0.09
1 vaccination+previous infection ≥12 months previously	0.59	0.57	0.60	0.10	0.08	0.13
2 vaccinations only	0.90	0.89	0.90	0.28	0.27	0.28
2 vaccinations+previous infection <6 months previously	0.40	0.39	0.41	0.05	0.04	0.07
2 vaccinations+previous infection 6–11 months previously	0.49	0.49	0.50	0.04	0.04	0.05
2 vaccinations+previous infection ≥12 months previously	0.62	0.61	0.63	0.06	0.06	0.07
3 vaccinations only	0.68	0.68	0.69	0.11	0.11	0.12
3 vaccinations+previous infection <6 months previously	0.22	0.21	0.23	0.01	0.01	0.02
3 vaccinations+previous infection >6 months previously	0.36	0.35	0.36	0.02	0.02	0.02

LCL, lower control limit; UCL, upper control limit.

### Waning in the NCC analysis

In the NCC, there were 8229 individuals with severe COVID-19 outcomes following primary infection in the Brazilian cohort and 246 individuals in the Scottish cohort (online supplemental table S6). These cases were matched with 657 488 and 826 726 controls, respectively.

Neither the Brazilian nor Scottish data demonstrated a linear trend in waning over time of protection against severe outcomes as time elapsed following primary infection (table 4). In the Brazilian data, using 120–200 days postprimary infection as a reference, the odds of severe outcome increased in the 401–600 day group (OR 1.16 (95% CI 1.07 to 1.25)). However, in the group with >600 days since primary infection, the odds of a severe outcome did not demonstrate further waning (OR 1.11 (95% CI 0.93 to 1.32)), although the number of severe outcomes observed in this group fell (n=160). The Scottish data demonstrated a significant waning of protection against severe outcomes beyond 600 days from primary infection (OR 2.20 (95% CI 1.34 to 3.59)), although again the number of events was small (n=31).

**Table 4:** Odds of severe COVID-19 outcomes by time elapsed since primary infection for Brazil and Scotland

Time elapsed since primary natural infection (days)	Brazil				Scotland			
	Events (n)	OR	LCL	UCL	Events (n)	OR	LCL	UCL
120–200	2358	1.00	–	–	65	1.00	–	–
201–400	4422	1.05	1.00	1.11	88	1.17	0.82	1.66
401–600	1289	1.16	1.07	1.25	63	1.11	0.75	1.62
601+	160	1.11	0.93	1.32	31	2.20	1.34	3.59

LCL, lower control limit; UCL, upper control limit.

## Discussion

Using national datasets to investigate waning of protection against SARS-CoV-2, our study found evidence of sustained protection against severe COVID-19 outcomes at least 12 months after primary infection with SARS-CoV-2 in unvaccinated individuals. The protection conferred by previous infection appeared comparable to that offered by vaccination. Against symptomatic infection, our Brazilian dataset demonstrated a pattern of waning protection over the 12-month period following primary SARS-CoV-2 infection, but this was not replicated in our Scottish dataset. Although some waning was observed, there remained substantial protection against symptomatic reinfection beyond 12 months for individuals with a history of previous infection. The results observed between Scottish and Brazilian data are in the context of different patterns of dominant circulating virus and different vaccination regimens, where Brazil used inactivated virus vaccines in addition to the viral vector and mRNA vaccines used in Scotland. Our findings are consistent with evidence from Brazil in the pre-Omicron era demonstrating the additional protective benefits against symptomatic and severe COVID-19 in those with evidence of previous SARS-CoV-2 infection.^19^

Our findings are supported by a 2023 systematic review and meta-analysis, which included data from 65 international studies (predominantly North American and European in origin). This demonstrated sustained protection against severe disease at 40 weeks postprimary infection, with protection of around 90% for ancestral, Alpha, Beta, Delta and Omicron variants.^11^ These estimates are comparable to our data for severe outcomes. Waning over time of protection was reported against symptomatic reinfection, with the estimates for protection against symptomatic reinfection at 10 months reflecting those observed in our Brazilian dataset (ancestral, Alpha, Beta and Delta: 78.4% (95% CI 56.1% to 90.5%), Omicron: 37.7% (95% CI 22.8% to 54.1%)). This review included limited data beyond 40 weeks postprimary infection, meaning our study adds to the existing literature by evidencing sustained high levels of protection against severe disease and protection against symptomatic reinfection for at least 600 days and 12 months postprimary SARS-CoV-2 infection, respectively.

Understanding the biological processes driving immunity also supports our findings, where humoral immunity (mediated through neutralising antibodies) and cellular immunity (mediated by T cells) adopt different roles in protecting against SARS-CoV-2 infection and severe disease, respectively.^20 21^ Evidence suggests mutation in the SARS-CoV-2 lineage has allowed adaptation in newer variants to evade neutralising antibodies^22^; however, T cell responses appear to be better preserved in variants exhibiting neutralising antibody escape, such as Omicron.^23^ These mechanisms explain the sustained protection against severe disease in the event of waning protection against reinfection observed in our data.

Hybrid immunity, where individuals have both history of natural infection and vaccination, appears to offer the greatest magnitude of protection against both symptomatic reinfection and severe outcomes in our data. A similar pattern of waning is observed when compared with those without vaccination, with evidence of sustained protection against severe outcomes and waning immunity against symptomatic infection at 12 months postprimary infection. Changes in the waning profile against symptomatic infection when the Omicron variant was the dominant circulating variant in Brazil suggest that natural immunity against infection with Omicron and its sublineages differs from historic variants, with previous infection appearing less protective against Omicron. However, it is reassuring that, despite an increased risk of symptomatic infection, immunity against severe outcomes is sustained beyond 12 months from primary infection and the magnitude of protective effects appears similar to the overall study period. Our findings are consistent with other studies which have found hybrid immunity to provide superior protection against reinfection than previous infection or vaccination alone.^24 25^ The increased effectiveness and more sustained duration of protection against both severe disease and symptomatic reinfection with hybrid immunity are compelling arguments for continued vaccination efforts to prevent serious disease complications and mortality, even if there is probable widespread immunity in the population resulting from periods of high incidence of SARS-CoV-2 infection.

The different patterns of disease severity and vaccine effectiveness with Omicron variants are well established, with less severe disease and reduced vaccine effectiveness against symptomatic infection observed.^26 27^ Systematic reviews investigating protection against reinfection with SARS-CoV-2 have demonstrated a different profile of immunity against Omicron variants when compared with earlier variants,^11 24^ in keeping with our findings. One systematic review, including 26 studies, found rapidly waning protection against symptomatic reinfection with Omicron variants, but sustained high protection against severe disease.^24^ Against severe disease at 12 months, effectiveness of previous infection alone was estimated as 74.6% (95% CI 63.1% to 83.5%) and of hybrid immunity effectiveness was 97.4% (95% CI 91.4% to 99.2%), demonstrating similar magnitude of protection and increased efficacy of hybrid immunity against Omicron reinfection as observed in our study. Given the difference in protective profile observed against Omicron variants, the protection conferred by Omicron infection against future reinfection warrants further research. Previous Omicron infection appears to provide better protection against reinfection with Omicron subvariants, compared with primary infection with pre-Omicron variants,^28^ although the magnitude of protection varies between Omicron sublineages.^29^ However, there is a scarcity of research describing the waning profile of post-Omicron infection against future reinfection. Lack of data describing the post-Omicron phase during our study period prevented our exploration of this issue, but it remains of policy importance. Future research should assess the role of Omicron and subsequent variants in evolving patterns of immunity and consider the duration of immunity over longer time periods from primary infection to reinfection.

Our study has strength in its use of harmonised analyses across differing international contexts, where vaccination schedules, test availability and dominant circulating variants differed throughout the study duration. The international consistency in our findings provides greater assurance in their validity. In particular, Brazil and Scotland had different approaches to testing during the COVID-19 pandemic, where the former implemented an approach with less testing and limited availability of tests during the Omicron surge in 2022. However, the similarities in results across the two countries, despite the differing testing strategies, reinforce the robustness of the findings. Using national databases, with near-complete population coverage, allows us to investigate clinically relevant outcomes and provides high statistical power. However, the sustained protection conferred by previous SARS-CoV-2 infection against severe outcomes, particularly in combination with vaccination, means that despite using a national dataset and study dates capturing the duration of universal testing in Scotland, the number of severe events occurring in the Scottish population was small, reducing the precision of our estimates. This important limitation must be noted when considering the implications of our findings.

Our study has several limitations. The waning of immunity conferred by vaccination may cause residual confounding in our results, as time elapsed since vaccination was not included in our analysis. Similarly, there is a correlation in the time elapsed since primary infection with changes in the dominant circulating variant, further contributing to the potential for residual confounding in our estimates of waning immunity. The introduction of survival bias was unavoidable as it was inherent that our study population must have survived their initial SARS-CoV-2 infection to be at risk of reinfection. There is potential for misclassification bias of individuals without documented history of SARS-CoV-2 infection if previous infections have been undetected, either through lack of testing availability (particularly early in the pandemic where restrictions on access to testing were enforced in some settings) or individual factors affecting decisions to seek COVID-19 testing. The potential impact of undocumented prior infections has been recognised as a limitation of using TND studies to investigate waning immunity, with misclassification found to underestimate effect estimates of reinfection. The magnitude of underestimation increases as the proportion of the population infected grows, meaning the potential for underestimation in our study is greater following the emergence of the Omicron variant. However, while use of TND design may result in underestimation in our effect estimates, it remains an appropriate choice of study design for our research question because of its strengths in minimising other potential biases (eg, differential testing and healthcare-seeking behaviours).^30^ Additionally, there is a potential bias in our ascertainment of severe outcomes, where individuals screened for COVID-19 as part of routine hospital admission protocols may be included in hospitalisation data, despite their SARS-CoV-2 infection being incidental to their reason for hospital admission. This will be particularly relevant during periods of high community incidence of COVID-19, as seen during the latter period of our study duration.

Reinfection was determined based on repeated positive test results and self-reported symptoms. Other observational studies investigating reinfection have applied a 90-day window between positive test results to identify reinfection; however, we adopted a more conservative 120-day window for determining reinfection. This approach may result in an underestimate of the odds of reinfection compared with studies using the 90-day threshold. Finally, in the Omicron period analysis, it was assumed that infections were due to Omicron variants as genotyping results were not available for all positive tests. This may have resulted in overascertainment of Omicron infections, as a result biasing our results in favour of better immunity against Omicron.

## Conclusions

The sustained duration of natural immunity against severe COVID-19 and the changes in waning profile against symptomatic infection to different viral variants have important policy implications for vaccination policy and the implementation of restrictions to reduce harm from COVID-19. Our findings are of significance to policymakers who should consider the role of waning of immunity from previous infection alongside immunity from vaccination when responding to the emergence of new SARS-CoV-2 variants and weighing the need for future vaccination. The protection conferred by previous SARS-CoV-2 infection against future severe disease must be weighed against the risks of primary infection of both mortality and morbidity, including complications during acute illness and long-term consequences of severe COVID-19. Vaccination provides an effective approach to confer protection against severe disease and symptomatic infection without these risks.

## Supplementary material

10.1136/bmjopen-2025-104057 online supplemental file 1

## Data Availability

Data may be obtained from a third party and are not publicly available.
