# Supplementary material for: Sustained natural immunity following SARS-CoV-2 infection against severe COVID-19 outcomes and symptomatic reinfection: analyses of national data for Brazil and Scotland
Source: BMJ Open. 2025 Jul 16;15(7):e104057. doi: 10.1136/bmjopen-2025-104057 (PMC12273164; doi:10.1136/bmjopen-2025-104057)
Supplement: online supplemental file 1 [file bmjopen-15-7-s001.docx]

**Additional Files**

S1 Figure: Data flow diagrams for test-negative design analysis.

S2 Figure: Data flow diagram for nested case control analysis.

S1 Table: STROBE checklists.

S2 Table: International Classification of Diseases-10 codes for Covid-19 illness.

S3 Table: Characteristics of the study population by vaccination and infection status for the Test Negative Design analysis in Brazil.

S4 Table: Characteristics of the study population by vaccination and infection status for the Test Negative Design analysis restricted to the post-Omicron phase in Brazil.

S5 Table: Test-negative design analysis demonstrating odds ratios (OR) and associated 95% confidence intervals (CI) for risk of symptomatic infection and severe disease outcomes by history of previous infection and vaccination status in Brazil during the pre-Omicron period.

S6 Table: Characteristics of data for nested case control analysis.

**S1 Figure**: Data flow diagrams demonstrating inclusion and exclusion criteria for TND analysis for a) Brazil and b) Scotland

| 1. **Brazil**   **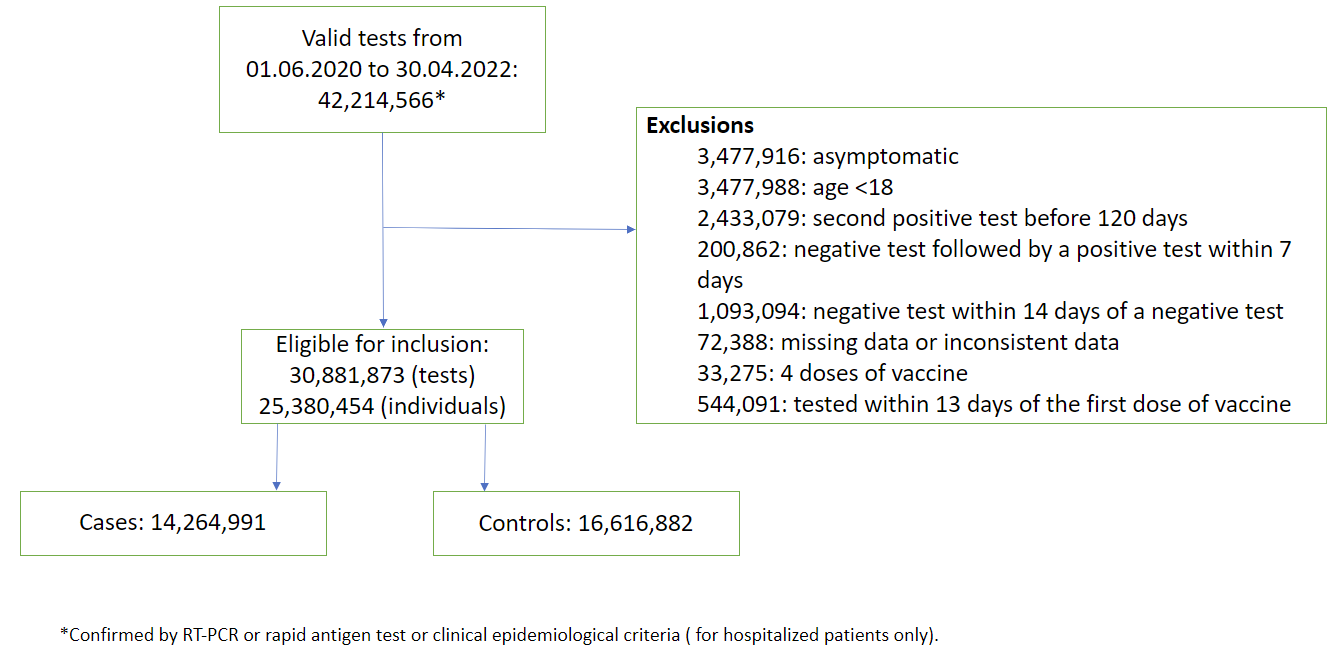** |
| --- |
| 1. **Scotland**   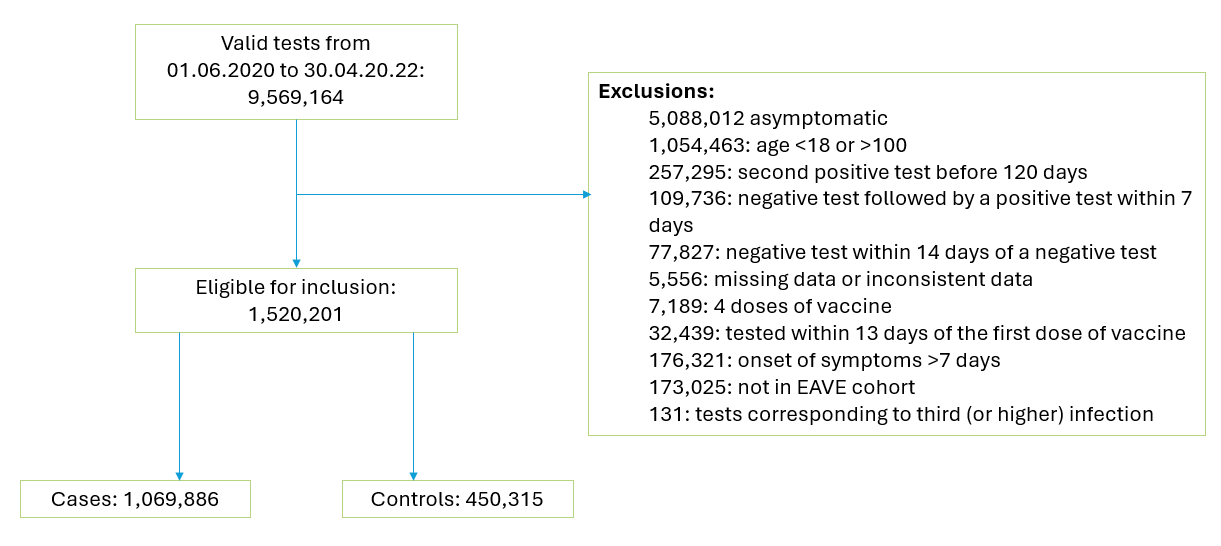 |

**S2 Figure:** Data flow diagrams demonstrating inclusion and exclusion criteria for NCC analysis for a) Brazil and b) Scotland

| 1. **Brazil**   **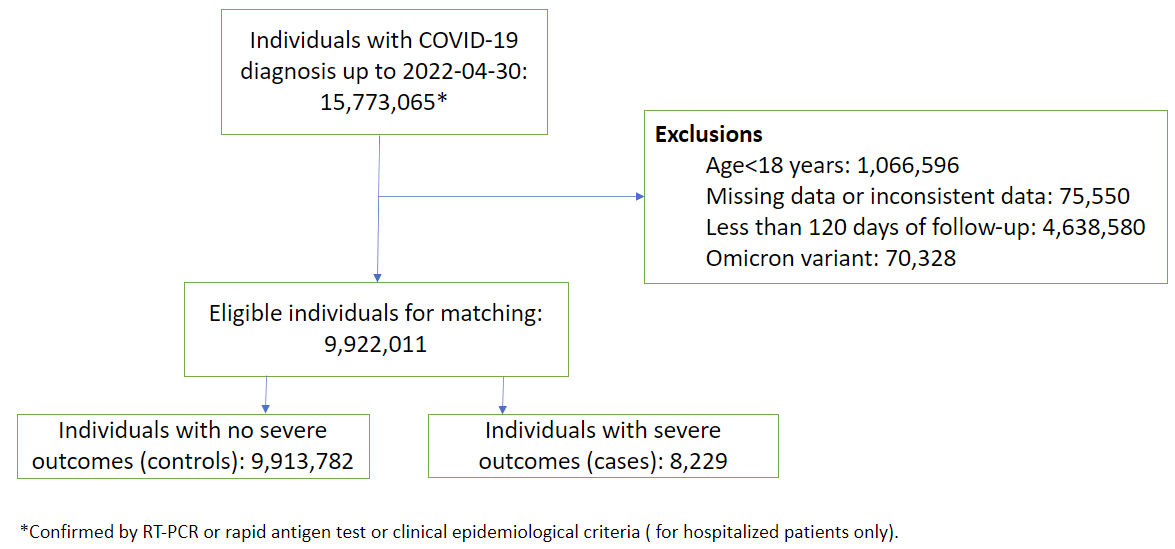** |
| --- |
| 1. **Scotland**   **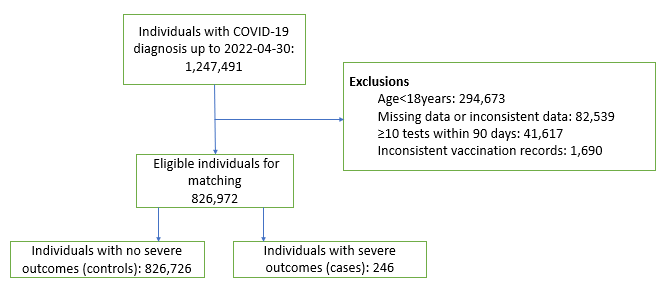** |

**S1 Table:** STROBE reporting checklists^1^

|  | **Item No.** | **STROBE items** | **Location in manuscript where items are reported** |
| --- | --- | --- | --- |
| Title and abstract | 1 | (a) Indicate the study’s design with a commonly used term in the title or the abstract (b) Provide in the abstract an informative and balanced summary of what was done and what was found | Abstract |
| Background rationale | 2 | Explain the scientific background and rationale for the investigation being reported | Introduction, paragraphs 1-3. |
| Objectives | 3 | State specific objectives, including any prespecified hypotheses | Introduction, paragraph 4. |
| Study design | 4 | Present key elements of study design early in the paper | Methods – Study design and variables. |
| Setting | 5 | Describe the setting, locations, and relevant dates, including periods of recruitment, exposure, follow-up, and data collection | Methods –Study design and variables. |
| Participants | 6 | (a) Cohort study - Give the eligibility criteria, and the sources and methods of selection of participants. Describe methods of follow-up  Case-control study - Give the eligibility criteria, and the sources and methods of case ascertainment and control selection. Give the rationale for the choice of cases and controls  Cross-sectional study - Give the eligibility criteria, and the sources and methods of selection of participants  (b) Cohort study - For matched studies, give matching criteria and number of exposed and unexposed  Case-control study - For matched studies, give matching criteria and the number of controls per case | Methods – Study design and variables, Table 1 and Supporting Information S1 and S2 Figures. |
| Variables | 7 | Clearly define all outcomes, exposures, predictors, potential confounders, and effect modifiers. Give diagnostic criteria, if applicable. | Methods– outcomes and exposures. |
| Data sources/ measurement | 8 | For each variable of interest, give sources of data and details of methods of assessment (measurement).  Describe comparability of assessment methods if there is more than one group | Methods – study design and variables. |
| Bias | 9 | Describe any efforts to address potential sources of bias | Methods – study design and variables; and discussion. |
| Study size | 10 | Explain how the study size was arrived at | Methods – study design and variables, and Supporting Information S1 and S2 Figures. |
| Quantitative variables | 11 | Explain how quantitative variables were handled in the analyses. If applicable, describe which groupings were chosen, and why | Methods – study design and variables. |
| Statistical methods | 12 | (a) Describe all statistical methods, including those used to control for confounding  (b) Describe any methods used to examine subgroups and interactions  (c) Explain how missing data were addressed  (d) Cohort study - If applicable, explain how loss to follow-up was addressed  Case-control study - If applicable, explain how matching of cases and controls was addressed  Cross-sectional study - If applicable, describe analytical methods taking account of sampling strategy  (e) Describe any sensitivity analyses | Methods – statistical analysis. |
| Participants | 13 | (a) Report the numbers of individuals at each stage of the study (e.g., numbers potentially eligible, examined for eligibility, confirmed eligible, included in the study, completing follow-up, and analysed)  (b) Give reasons for non-participation at each stage.  (c) Consider use of a flow diagram | Table 1 and Supporting Information: S1 and S2 figures, and S3, S4 and S6 tables. |
| Descriptive data | 14 | (a) Give characteristics of study participants (e.g., demographic, clinical, social) and information on exposures and potential confounders  (b) Indicate the number of participants with missing data for each variable of interest  (c) Cohort study - summarise follow-up time (e.g., average and total amount) | Table 1 and Supporting Information S3, S4 and S6 tables. |
| Outcome data | 15 | Cohort study - Report numbers of outcome events or summary measures over time  Case-control study - Report numbers in each exposure category, or summary measures of exposure  Cross-sectional study - Report numbers of outcome events or summary measures | Results, tables 2-4 and supplementary materials (S5 table). |
| Main results | 16 | (a) Give unadjusted estimates and, if applicable, confounder-adjusted estimates and their precision (e.g., 95% confidence interval). Make clear which confounders were adjusted for and why they were included  (b) Report category boundaries when continuous variables were categorized  (c) If relevant, consider translating estimates of relative risk into absolute risk for a meaningful time period | Results, tables 2 – 4 and supplementary materials (S5 table). |
| Other analyses | 17 | Report other analyses done—e.g., analyses of subgroups and interactions, and sensitivity analyses | Supporting Information. |
| Key results | 18 | Summarise key results with reference to study objectives | Discussion, paragraph 1. |
| Limitations | 19 | Discuss limitations of the study, taking into account sources of potential bias or imprecision. Discuss both direction and magnitude of any potential bias | Discussion, paragraphs 6 and 7. |
| Interpretation | 20 | Give a cautious overall interpretation of results considering objectives, limitations, multiplicity of analyses, results from similar studies, and other relevant evidence | Discussion, paragraphs 2 – 5. |
| Generalisability | 21 | Discuss the generalisability (external validity) of the study results | Discussion, paragraph 5. |
| Funding | 22 | Give the source of funding and the role of the funders for the present study and, if applicable, for the original study on which the present article is based | Acknowledgements |

Reference:

1. Benchimol EI, Smeeth L, Guttmann A, et al. The REporting of studies Conducted using Observational Routinely-collected health Data (RECORD) Statement. PLOS Medicine 2015;12:e1001885.

**S2 Table**: International Classification of Diseases-10 codes for Covid-19 illness.

| **Code** | **Description** |
| --- | --- |
| U07.1 | Covid-19, virus identified |
| U07.2 | Covid-19, virus not identified |
| Source: <https://www.who.int/classifications/icd/COVID-19-coding-icd10.pdf> | |

**S3 Table: Characteristics of the study population by vaccination and infection status for the Test Negative Design analysis in Brazil.**

| **Characteristic** | **Unvaccinated** | **Only natural infection <6 months previously** | **Only natural infection 6 – 11 months previously** | **Only natural infection ≥12 months previously** | **1 vaccination only** | **1 vaccination + previous infection <6 months previously** | **1 vaccination + previous infection 6-11 months previously** | **1 vaccination + previous infection ≥12 months previously** | **2 vaccinations only** | **2 vaccinations + previous infection <6 months previously** | **2 vaccinations + previous infection 6-11 months previously** | **2 vaccinations + previous infection ≥12 months previously** | **3 vaccinations only** | **3 vaccinations + previous infection <6 months previously** | **3 vaccinations + previous infection >6 months previously** |
| --- | --- | --- | --- | --- | --- | --- | --- | --- | --- | --- | --- | --- | --- | --- | --- |
| **N** | 16,657,485 | 80,872 | 20,786 | 132,128 | 3,252,682 | 44,015 | 44,741 | 115,138 | 7,490,224 | 63,956 | 235,637 | 380,893 | 2,155,678 | 9,943 | N = 197,6951 |
| **Age**  (median; Q1,Q3) | 38 (28, 50) | 34 (26, 43) | 35 (28, 44) | 35 (27, 44) | 40 (30, 52) | 38 (29, 47) | 36 (28, 44) | 37 (28, 45) | 39 (29, 52) | 37 (28, 49) | 37 (29, 46) | 37 (28, 47) | 53 (38, 67) | 47 (35, 64) | 44 (34, 57) |
| **Sex: Female** | 8,767,416 (52.6%) | 46,066 (57.0%) | 10,863 (52.3%) | 73,523 (55.6%) | 1,857,935 (57.1%) | 26,574 (60.4%) | 25,531 (57.1%) | 67,630 (58.7%) | 4,464,189 (59.6%) | 40,366 (63.1%) | 142,478 (60.5%) | 231,797 (60.9%) | 1,391,215 (64.5%) | 6,849 (68.9%) | 134,643 (68.1%) |
| **Ethnicity** | | | | | | | | | | | | | | | |
| White | 7,732,069 (46.4%) | 47,113 (58.3%) | 11,897 (57.2%) | 75,787 (57.4%) | 1,405,394 (43.2%) | 24,945 (56.7%) | 25,127 (56.2%) | 66,623 (57.9%) | 3,252,895 (43.4%) | 35,505 (55.5%) | 139,019 (59.0%) | 220,458 (57.9%) | 1,024,310 (47.5%) | 5,650 (56.8%) | 118,240 (59.8%) |
| Black | 685,586 (4.1%) | 3,120 (3.9%) | 765 (3.7%) | 5,113 (3.9%) | 131,926 (4.1%) | 1,715 (3.9%) | 1,819 (4.1%) | 4,399 (3.8%) | 290,765 (3.9%) | 2,535 (4.0%) | 9,191 (3.9%) | 14,710 (3.9%) | 74,362 (3.4%) | 384 (3.9%) | 7,633 (3.9%) |
| Asian | 442,784 (2.7%) | 1,396 (1.7%) | 595 (2.9%) | 3,047 (2.3%) | 46,438 (1.4%) | 544 (1.2%) | 1,271 (2.8%) | 1,882 (1.6%) | 131,124 (1.8%) | 1,026 (1.6%) | 5,648 (2.4%) | 6,301 (1.7%) | 35,022 (1.6%) | 190 (1.9%) | 3,558 (1.8%) |
| Mixed | 5,033,402 (30.2%) | 23,316 (28.8%) | 6,466 (31.1%) | 41,331 (31.3%) | 1,027,152 (31.6%) | 12,850 (29.2%) | 14,323 (32.0%) | 33,264 (28.9%) | 2,294,604 (30.6%) | 19,228 (30.1%) | 67,840 (28.8%) | 111,117 (29.2%) | 588,577 (27.3%) | 2,861 (28.8%) | 56,710 (28.7%) |
| Indigenous | 15,816 (0.1%) | 29 (0.0%) | 40 (0.2%) | 94 (0.1%) | 3,391 (0.1%) | 26 (0.1%) | 32 (0.1%) | 59 (0.1%) | 14,253 (0.2%) | 59 (0.1%) | 233 (0.1%) | 292 (0.1%) | 4,425 (0.2%) | 35 (0.4%) | 308 (0.2%) |
| Missing | 2,747,828 (16.5%) | 5,898 (7.3%) | 1,023 (4.9%) | 6,756 (5.1%) | 638,381 (19.6%) | 3,935 (8.9%) | 2,169 (4.8%) | 8,911 (7.7%) | 1,506,583 (20.1%) | 5,603 (8.8%) | 13,706 (5.8%) | 28,015 (7.4%) | 428,982 (19.9%) | 823 (8.3%) | 11,246 (5.7%) |
| **Age group** | | | | | | | | | | | | | | | |
| 18-59 years | 14,720,142 (88.4%) | 77,004 (95.2%) | 19,577 (94.2%) | 125,971 (95.3%) | 2,772,753 (85.2%) | 40,761 (92.6%) | 43,201 (96.6%) | 109,183 (94.8%) | 6,342,941 (84.7%) | 56,759 (88.7%) | 220,467 (93.6%) | 351,650 (92.3%) | 1,320,555 (61.3%) | 6,850 (68.9%) | 155,491 (78.7%) |
| >59 years | 1,937,343 (11.6%) | 3,868 (4.8%) | 1,209 (5.8%) | 6,157 (4.7%) | 479,929 (14.8%) | 3,254 (7.4%) | 1,540 (3.4%) | 5,955 (5.2%) | 1,147,283 (15.3%) | 7,197 (11.3%) | 15,170 (6.4%) | 29,243 (7.7%) | 835,123 (38.7%) | 3,093 (31.1%) | 42,204 (21.3%) |
| **Test type** | | | | | | | | | | | | | | | |
| Antígen | 5,545,603 (33.3%) | 35,523 (43.9%) | 13,935 (67.0%) | 61,880 (46.8%) | 1,987,465 (61.1%) | 25,627 (58.2%) | 30,569 (68.3%) | 72,713 (63.2%) | 5,500,165 (73.4%) | 42,674 (66.7%) | 174,834 (74.2%) | 277,767 (72.9%) | 1,757,917 (81.5%) | 7,832 (78.8%) | 157,228 (79.5%) |
| RT-PCR | 11,111,882 (66.7%) | 45,349 (56.1%) | 6,851 (33.0%) | 70,248 (53.2%) | 1,265,217 (38.9%) | 18,388 (41.8%) | 14,172 (31.7%) | 42,425 (36.8%) | 1,990,059 (26.6%) | 21,282 (33.3%) | 60,803 (25.8%) | 103,126 (27.1%) | 397,761 (18.5%) | 2,111 (21.2%) | 40,467 (20.5%) |
| **Diabetes** | 703,534 (4.2%) | 2,937 (3.6%) | 743 (3.6%) | 5,031 (3.8%) | 145,441 (4.5%) | 2,154 (4.9%) | 1,608 (3.6%) | 4,521 (3.9%) | 270,767 (3.6%) | 3,395 (5.3%) | 10,232 (4.3%) | 16,507 (4.3%) | 125,094 (5.8%) | 1,014 (10.2%) | 16,581 (8.4%) |
| **Obesity** | 285,610 (1.7%) | 1,769 (2.2%) | 397 (1.9%) | 2,786 (2.1%) | 51,894 (1.6%) | 1,240 (2.8%) | 823 (1.8%) | 2,754 (2.4%) | 82,162 (1.1%) | 1,740 (2.7%) | 4,769 (2.0%) | 9,286 (2.4%) | 25,360 (1.2%) | 332 (3.3%) | 5,823 (2.9%) |
| **Immunosuppression** | 137,277 (0.8%) | 949 (1.2%) | 282 (1.4%) | 1,599 (1.2%) | 25,843 (0.8%) | 570 (1.3%) | 468 (1.0%) | 1,191 (1.0%) | 45,707 (0.6%) | 728 (1.1%) | 2,371 (1.0%) | 3,484 (0.9%) | 22,536 (1.0%) | 207 (2.1%) | 3,465 (1.8%) |
| **Chronic respiratory disease** | 537,088 (3.2%) | 3,990 (4.9%) | 963 (4.6%) | 6,722 (5.1%) | 99,401 (3.1%) | 2,274 (5.2%) | 2,139 (4.8%) | 5,307 (4.6%) | 215,726 (2.9%) | 3,358 (5.3%) | 11,095 (4.7%) | 17,640 (4.6%) | 70,200 (3.3%) | 621 (6.2%) | 11,164 (5.6%) |
| **Cardiac disease** | 1,335,515 (8.0%) | 5,984 (7.4%) | 1,487 (7.2%) | 10,215 (7.7%) | 256,476 (7.9%) | 4,230 (9.6%) | 3,161 (7.1%) | 9,077 (7.9%) | 471,466 (6.3%) | 6,398 (10.0%) | 19,639 (8.3%) | 31,868 (8.4%) | 198,316 (9.2%) | 1,728 (17.4%) | 29,142 (14.7%) |
| **Chronic kidney Disease** | 89,008 (0.5%) | 471 (0.6%) | 140 (0.7%) | 801 (0.6%) | 16,161 (0.5%) | 276 (0.6%) | 237 (0.5%) | 555 (0.5%) | 28,977 (0.4%) | 361 (0.6%) | 1,190 (0.5%) | 1,698 (0.4%) | 12,947 (0.6%) | 100 (1.0%) | 1,769 (0.9%) |
| **Number of comorbidities** | | | | | | | | | | | | | | | |
| 0 | 14,322,717 (86.0%) | 68,562 (84.8%) | 17,723 (85.3%) | 111,310 (84.2%) | 2,806,355 (86.3%) | 35,945 (81.7%) | 38,146 (85.3%) | 96,944 (84.2%) | 6,644,297 (88.7%) | 52,133 (81.5%) | 197,595 (83.9%) | 319,220 (83.8%) | 1,813,995 (84.1%) | 7,105 (71.5%) | 148,347 (75.0%) |
| 1 | 1,719,809 (10.3%) | 9,329 (11.5%) | 2,334 (11.2%) | 15,845 (12.0%) | 325,201 (10.0%) | 5,949 (13.5%) | 5,150 (11.5%) | 14,019 (12.2%) | 626,542 (8.4%) | 8,538 (13.3%) | 29,039 (12.3%) | 46,588 (12.2%) | 248,273 (11.5%) | 1,931 (19.4%) | 34,466 (17.4%) |
| 2 | 496,013 (3.0%) | 2,321 (2.9%) | 546 (2.6%) | 3,858 (2.9%) | 97,234 (3.0%) | 1,651 (3.8%) | 1,117 (2.5%) | 3,315 (2.9%) | 177,079 (2.4%) | 2,568 (4.0%) | 7,097 (3.0%) | 11,949 (3.1%) | 76,653 (3.6%) | 692 (7.0%) | 11,766 (6.0%) |
| 3+ | 118,946 (0.7%) | 660 (0.8%) | 183 (0.9%) | 1,115 (0.8%) | 23,892 (0.7%) | 470 (1.1%) | 328 (0.7%) | 860 (0.7%) | 42,306 (0.6%) | 717 (1.1%) | 1,906 (0.8%) | 3,136 (0.8%) | 16,757 (0.8%) | 215 (2.2%) | 3,116 (1.6%) |
| **Test Result** | | | | | | | | | | | | | | | |
| Negative | 8,643,064 (51.9%) | 68,929 (85.2%) | 13,722 (66.0%) | 107,660 (81.5%) | 2,004,195 (61.6%) | 39,866 (90.6%) | 31,686 (70.8%) | 94,286 (81.9%) | 3,897,375 (52.0%) | 51,414 (80.4%) | 139,861 (59.4%) | 255,379 (67.0%) | 1,126,016 (52.2%) | 7,838 (78.8%) | 135,591 (68.6%) |
| Positive | 8,014,421 (48.1%) | 11,943 (14.8%) | 7,064 (34.0%) | 24,468 (18.5%) | 1,248,487 (38.4%) | 4,149 (9.4%) | 13,055 (29.2%) | 20,852 (18.1%) | 3,592,849 (48.0%) | 12,542 (19.6%) | 95,776 (40.6%) | 125,514 (33.0%) | 1,029,662 (47.8%) | 2,105 (21.2%) | 62,104 (31.4%) |
| **First vaccine type** | | | | | | | | | | | | | | | |
| Ad26 | 0 (NA%) | 0 (NA%) | 0 (NA%) | 0 (NA%) | 275,812 (8.5%) | 3,224 (7.3%) | 8,511 (19.0%) | 14,848 (12.9%) | 118,986 (1.6%) | 569 (0.9%) | 5,977 (2.5%) | 7,997 (2.1%) | 108 (0.0%) | 1 (0.0%) | 15 (0.0%) |
| AZ | 0 (NA%) | 0 (NA%) | 0 (NA%) | 0 (NA%) | 1,546,889 (47.6%) | 20,969 (47.6%) | 14,458 (32.3%) | 45,310 (39.4%) | 3,051,444 (40.7%) | 23,591 (36.9%) | 101,517 (43.1%) | 153,039 (40.2%) | 954,739 (44.3%) | 3,722 (37.4%) | 83,875 (42.4%) |
| BNT162b2 | 0 (NA%) | 0 (NA%) | 0 (NA%) | 0 (NA%) | 854,612 (26.3%) | 13,410 (30.5%) | 14,871 (33.2%) | 36,853 (32.0%) | 1,825,856 (24.4%) | 13,261 (20.7%) | 65,558 (27.8%) | 106,306 (27.9%) | 156,539 (7.3%) | 405 (4.1%) | 16,730 (8.5%) |
| CV | 0 (NA%) | 0 (NA%) | 0 (NA%) | 0 (NA%) | 575,369 (17.7%) | 6,412 (14.6%) | 6,901 (15.4%) | 18,127 (15.7%) | 2,493,938 (33.3%) | 26,535 (41.5%) | 62,585 (26.6%) | 113,551 (29.8%) | 1,044,292 (48.4%) | 5,815 (58.5%) | 97,075 (49.1%) |
| Unknown | 16,657,485 | 80,872 | 20,786 | 132,128 | 0 | 0 | 0 | 0 | 0 | 0 | 0 | 0 | 0 | 0 | 0 |
| **Second vaccine type** | | | | | | | | | | | | | | | |
| Ad26 | 0 (NA%) | 0 (NA%) | 0 (NA%) | 0 (NA%) | 0 (NA%) | 0 (NA%) | 0 (NA%) | 0 (NA%) | 95,104 (1.3%) | 447 (0.7%) | 5,000 (2.1%) | 6,607 (1.7%) | 142 (0.0%) | 0 (0.0%) | 12 (0.0%) |
| AZ | 0 (NA%) | 0 (NA%) | 0 (NA%) | 0 (NA%) | 0 (NA%) | 0 (NA%) | 0 (NA%) | 0 (NA%) | 2,887,511 (38.6%) | 22,377 (35.0%) | 95,616 (40.6%) | 144,956 (38.1%) | 943,149 (43.8%) | 3,685 (37.1%) | 82,584 (41.8%) |
| BNT162b2 | 0 (NA%) | 0 (NA%) | 0 (NA%) | 0 (NA%) | 0 (NA%) | 0 (NA%) | 0 (NA%) | 0 (NA%) | 2,050,264 (27.4%) | 14,846 (23.2%) | 74,236 (31.5%) | 117,167 (30.8%) | 168,222 (7.8%) | 445 (4.5%) | 17,991 (9.1%) |
| CV | 0 (NA%) | 0 (NA%) | 0 (NA%) | 0 (NA%) | 0 (NA%) | 0 (NA%) | 0 (NA%) | 0 (NA%) | 2,457,345 (32.8%) | 26,286 (41.1%) | 60,785 (25.8%) | 112,163 (29.4%) | 1,044,165 (48.4%) | 5,813 (58.5%) | 97,108 (49.1%) |
| Unknown | 16,657,485 | 80,872 | 20,786 | 132,128 | 3,252,682 | 44,015 | 44,741 | 115,138 | 0 | 0 | 0 | 0 | 0 | 0 | 0 |
| **Third vaccine type** | | | | | | | | | | | | | | | |
| Ad26 | 0 (NA%) | 0 (NA%) | 0 (NA%) | 0 (NA%) | 0 (NA%) | 0 (NA%) | 0 (NA%) | 0 (NA%) | 0 (NA%) | 0 (NA%) | 0 (NA%) | 0 (NA%) | 20,030 (0.9%) | 83 (0.8%) | 2,287 (1.2%) |
| AZ | 0 (NA%) | 0 (NA%) | 0 (NA%) | 0 (NA%) | 0 (NA%) | 0 (NA%) | 0 (NA%) | 0 (NA%) | 0 (NA%) | 0 (NA%) | 0 (NA%) | 0 (NA%) | 93,187 (4.3%) | 363 (3.7%) | 8,706 (4.4%) |
| BNT162b2 | 0 (NA%) | 0 (NA%) | 0 (NA%) | 0 (NA%) | 0 (NA%) | 0 (NA%) | 0 (NA%) | 0 (NA%) | 0 (NA%) | 0 (NA%) | 0 (NA%) | 0 (NA%) | 1,923,103 (89.2%) | 9,091 (91.4%) | 178,342 (90.2%) |
| CV | 0 (NA%) | 0 (NA%) | 0 (NA%) | 0 (NA%) | 0 (NA%) | 0 (NA%) | 0 (NA%) | 0 (NA%) | 0 (NA%) | 0 (NA%) | 0 (NA%) | 0 (NA%) | 119,358 (5.5%) | 406 (4.1%) | 8,360 (4.2%) |
| Unknown | 16,657,485 | 80,872 | 20,786 | 132,128 | 3,252,682 | 44,015 | 44,741 | 115,138 | 7,490,224 | 63,956 | 235,637 | 380,893 | 0 | 0 | 0 |
| **Hospitalization** | 1,154,979 (6.9%) | 1,426 (1.8%) | 371 (1.8%) | 2,151 (1.6%) | 157,470 (4.8%) | 552 (1.3%) | 422 (0.9%) | 1,138 (1.0%) | 229,408 (3.1%) | 860 (1.3%) | 2,079 (0.9%) | 3,048 (0.8%) | 66,215 (3.1%) | 249 (2.5%) | 2,575 (1.3%) |
| **Death** | 310,873 (1.9%) | 293 (0.4%) | 80 (0.4%) | 467 (0.4%) | 50,351 (1.5%) | 139 (0.3%) | 80 (0.2%) | 237 (0.2%) | 74,000 (1.0%) | 189 (0.3%) | 375 (0.2%) | 593 (0.2%) | 19,214 (0.9%) | 51 (0.5%) | 427 (0.2%) |
| **Severe outcome** | 1,199,316 (7.2%) | 1,496 (1.8%) | 391 (1.9%) | 2,235 (1.7%) | 164,697 (5.1%) | 579 (1.3%) | 441 (1.0%) | 1,192 (1.0%) | 240,024 (3.2%) | 905 (1.4%) | 2,169 (0.9%) | 3,176 (0.8%) | 69,139 (3.2%) | 257 (2.6%) | 2,669 (1.4%) |

Ad26: Ad26.COV2.S vaccine; AZ: ChAdOx1 nCoV-19 vaccine; BNT162b2: BNT162b2 vaccine; CV: CoronaVac vaccine; RT-PCR: reverse transcription polymerase chain reaction.

**S4 Table:** **Characteristics of the study population by vaccination and infection status for the Test Negative Design analysis restricted to the post-Omicron phase in Brazil.**

| **Characteristic** | **Unvaccinated** | **Only natural infection <6 months previously** | **Only natural infection 6 – 11 months previously** | **Only natural infection ≥12 months previously** | **1 vaccination only** | **1 vaccination + previous infection <6 months previously** | **1 vaccination + previous infection 6-11 months previously** | **1 vaccination + previous infection ≥12 months previously** | **2 vaccinations only** | **2 vaccinations + previous infection <6 months previously** | **2 vaccinations + previous infection 6-11 months previously** | **2 vaccinations + previous infection ≥12 months previously** | **3 vaccinations only** | **3 vaccinations + previous infection <6 months previously** | **3 vaccinations + previous infection >6 months previously** |
| --- | --- | --- | --- | --- | --- | --- | --- | --- | --- | --- | --- | --- | --- | --- | --- |
| **N** | 336,468 | 2,074 | 13,810 | 10,418 | 758,042 | 5,008 | 36,771 | 24,988 | 4,969,080 | 26,699 | 258,873 | 187,762 | 2,025,351 | 8,272 | 184,661 |
| **Age**  (median; Q1,Q3) | 37 (27, 49) | 34 (26, 45) | 36 (28, 47) | 38 (30, 48) | 34 (25, 43) | 31 (24, 40) | 35 (26, 42) | 36 (27, 43) | 37 (27, 47) | 34 (26, 44) | 36 (27, 45) | 36 (28, 46) | 52 (38, 66) | 46 (34, 62) | 44 (34, 57) |
| **Sex: Female** | 161,234 (47.9%) | 1,051 (50.7%) | 7,082 (51.3%) | 5,489 (52.7%) | 404,460 (53.4%) | 2,896 (57.8%) | 20,439 (55.6%) | 14,313 (57.3%) | 2,902,585 (58.4%) | 16,324 (61.1%) | 153,642 (59.4%) | 112,443 (59.9%) | 1,306,154 (64.5%) | 5,670 (68.5%) | 125,551 (68.0%) |
| **Ethnicity** | | | | | | | | | | | | | | | |
| White | 133,395 (39.6%) | 1,123 (54.1%) | 7,713 (55.9%) | 6,382 (61.3%) | 279,786 (36.9%) | 2,542 (50.8%) | 19,238 (52.3%) | 13,996 (56.0%) | 2,068,088 (41.6%) | 13,914 (52.1%) | 147,085 (56.8%) | 110,982 (59.1%) | 962,193 (47.5%) | 4,688 (56.7%) | 110,774 (60.0%) |
| Black | 12,571 (3.7%) | 75 (3.6%) | 511 (3.7%) | 318 (3.1%) | 34,225 (4.5%) | 215 (4.3%) | 1,576 (4.3%) | 1,020 (4.1%) | 195,857 (3.9%) | 1,120 (4.2%) | 10,119 (3.9%) | 7,272 (3.9%) | 69,666 (3.4%) | 326 (3.9%) | 7,080 (3.8%) |
| Asian | 6,550 (1.9%) | 41 (2.0%) | 258 (1.9%) | 243 (2.3%) | 16,811 (2.2%) | 112 (2.2%) | 724 (2.0%) | 672 (2.7%) | 99,898 (2.0%) | 559 (2.1%) | 4,521 (1.7%) | 4,519 (2.4%) | 33,291 (1.6%) | 170 (2.1%) | 3,306 (1.8%) |
| Mixed | 114,419 (34.0%) | 657 (31.7%) | 4,345 (31.5%) | 2,870 (27.5%) | 272,165 (35.9%) | 1,718 (34.3%) | 12,548 (34.1%) | 7,839 (31.4%) | 1,578,456 (31.8%) | 8,750 (32.8%) | 78,431 (30.3%) | 53,378 (28.4%) | 553,439 (27.3%) | 2,375 (28.7%) | 52,723 (28.6%) |
| Indigenous | 999 (0.3%) | 3 (0.1%) | 18 (0.1%) | 21 (0.2%) | 1,113 (0.1%) | 4 (0.1%) | 17 (0.0%) | 24 (0.1%) | 5,647 (0.1%) | 23 (0.1%) | 122 (0.0%) | 143 (0.1%) | 4,129 (0.2%) | 32 (0.4%) | 286 (0.2%) |
| Missing | 68,534 (20.4%) | 175 (8.4%) | 965 (7.0%) | 584 (5.6%) | 153,942 (20.3%) | 417 (8.3%) | 2,668 (7.3%) | 1,437 (5.8%) | 1,021,134 (20.5%) | 2,333 (8.7%) | 18,595 (7.2%) | 11,468 (6.1%) | 402,633 (19.9%) | 681 (8.2%) | 10,492 (5.7%) |
| **Age group** | | | | | | | | | | | | | | | |
| 18-59 years | 294,178 (87.4%) | 1,915 (92.3%) | 12,785 (92.6%) | 9,533 (91.5%) | 724,532 (95.6%) | 4,890 (97.6%) | 35,710 (97.1%) | 24,257 (97.1%) | 4,596,371 (92.5%) | 25,563 (95.7%) | 246,733 (95.3%) | 178,126 (94.9%) | 1,262,636 (62.3%) | 5,848 (70.7%) | 146,349 (79.3%) |
| >59 years | 42,290 (12.6%) | 159 (7.7%) | 1,025 (7.4%) | 885 (8.5%) | 33,510 (4.4%) | 118 (2.4%) | 1,061 (2.9%) | 731 (2.9%) | 372,709 (7.5%) | 1,136 (4.3%) | 12,140 (4.7%) | 9,636 (5.1%) | 762,715 (37.7%) | 2,424 (29.3%) | 38,312 (20.7%) |
| **Test type** | | | | | | | | | | | | | | | |
| Antígen | 276,102 (82.1%) | 1,716 (82.7%) | 11,279 (81.7%) | 8,616 (82.7%) | 628,121 (82.9%) | 4,100 (81.9%) | 29,779 (81.0%) | 20,180 (80.8%) | 4,099,326 (82.5%) | 21,896 (82.0%) | 210,066 (81.1%) | 149,944 (79.9%) | 1,678,831 (82.9%) | 6,809 (82.3%) | 149,830 (81.1%) |
| RT-PCR | 60,366 (17.9%) | 358 (17.3%) | 2,531 (18.3%) | 1,802 (17.3%) | 129,921 (17.1%) | 908 (18.1%) | 6,992 (19.0%) | 4,808 (19.2%) | 869,754 (17.5%) | 4,803 (18.0%) | 48,807 (18.9%) | 37,818 (20.1%) | 346,520 (17.1%) | 1,463 (17.7%) | 34,831 (18.9%) |
| **Diabetes** | 7,808 (2.3%) | 64 (3.1%) | 409 (3.0%) | 396 (3.8%) | 10,029 (1.3%) | 102 (2.0%) | 893 (2.4%) | 645 (2.6%) | 99,484 (2.0%) | 806 (3.0%) | 8,503 (3.3%) | 6,915 (3.7%) | 113,394 (5.6%) | 789 (9.5%) | 15,212 (8.2%) |
| **Obesity** | 2,607 (0.8%) | 48 (2.3%) | 286 (2.1%) | 210 (2.0%) | 4,824 (0.6%) | 87 (1.7%) | 744 (2.0%) | 393 (1.6%) | 37,008 (0.7%) | 527 (2.0%) | 5,597 (2.2%) | 3,478 (1.9%) | 23,296 (1.2%) | 269 (3.3%) | 5,414 (2.9%) |
| **Immunosuppression** | 2,280 (0.7%) | 25 (1.2%) | 155 (1.1%) | 126 (1.2%) | 3,059 (0.4%) | 36 (0.7%) | 237 (0.6%) | 199 (0.8%) | 20,703 (0.4%) | 204 (0.8%) | 1,861 (0.7%) | 1,610 (0.9%) | 20,183 (1.0%) | 158 (1.9%) | 3,132 (1.7%) |
| **Chronic respiratory disease** | 7,849 (2.3%) | 75 (3.6%) | 551 (4.0%) | 424 (4.1%) | 17,411 (2.3%) | 217 (4.3%) | 1,544 (4.2%) | 1,019 (4.1%) | 115,754 (2.3%) | 1,105 (4.1%) | 10,608 (4.1%) | 8,098 (4.3%) | 63,441 (3.1%) | 483 (5.8%) | 10,202 (5.5%) |
| **Cardiac disease** | 14,171 (4.2%) | 137 (6.6%) | 899 (6.5%) | 769 (7.4%) | 18,657 (2.5%) | 217 (4.3%) | 1,928 (5.2%) | 1,329 (5.3%) | 171,582 (3.5%) | 1,581 (5.9%) | 16,936 (6.5%) | 13,519 (7.2%) | 177,662 (8.8%) | 1,364 (16.5%) | 26,756 (14.5%) |
| **Chronic kidney Disease** | 1,136 (0.3%) | 4 (0.2%) | 65 (0.5%) | 69 (0.7%) | 1,467 (0.2%) | 18 (0.4%) | 106 (0.3%) | 97 (0.4%) | 10,539 (0.2%) | 96 (0.4%) | 897 (0.3%) | 773 (0.4%) | 11,378 (0.6%) | 70 (0.8%) | 1,578 (0.9%) |
| **Number of comorbidities** | | | | | | | | | | | | | | | |
| 0 | 308,437 (91.7%) | 1,814 (87.5%) | 11,971 (86.7%) | 8,888 (85.3%) | 711,287 (93.8%) | 4,459 (89.0%) | 32,293 (87.8%) | 21,991 (88.0%) | 4,596,717 (92.5%) | 23,301 (87.3%) | 223,480 (86.3%) | 160,526 (85.5%) | 1,715,343 (84.7%) | 5,999 (72.5%) | 139,149 (75.4%) |
| 1 | 21,560 (6.4%) | 192 (9.3%) | 1,425 (10.3%) | 1,159 (11.1%) | 39,481 (5.2%) | 448 (8.9%) | 3,671 (10.0%) | 2,436 (9.7%) | 303,140 (6.1%) | 2,664 (10.0%) | 27,994 (10.8%) | 21,377 (11.4%) | 227,402 (11.2%) | 1,586 (19.2%) | 32,035 (17.3%) |
| 2 | 5,302 (1.6%) | 51 (2.5%) | 321 (2.3%) | 289 (2.8%) | 6,080 (0.8%) | 79 (1.6%) | 666 (1.8%) | 452 (1.8%) | 57,637 (1.2%) | 578 (2.2%) | 6,024 (2.3%) | 4,744 (2.5%) | 68,032 (3.4%) | 536 (6.5%) | 10,704 (5.8%) |
| 3+ | 1,169 (0.3%) | 17 (0.8%) | 93 (0.7%) | 82 (0.8%) | 1,194 (0.2%) | 22 (0.4%) | 141 (0.4%) | 109 (0.4%) | 11,586 (0.2%) | 156 (0.6%) | 1,375 (0.5%) | 1,115 (0.6%) | 14,574 (0.7%) | 151 (1.8%) | 2,773 (1.5%) |
| **Test Result** | | | | | | | | | | | | | | | |
| Negative | 128,637 (38.2%) | 1,176 (56.7%) | 6,724 (48.7%) | 4,697 (45.1%) | 326,858 (43.1%) | 3,165 (63.2%) | 20,377 (55.4%) | 13,184 (52.8%) | 2,005,584 (40.4%) | 16,267 (60.9%) | 140,857 (54.4%) | 95,675 (51.0%) | 1,008,603 (49.8%) | 6,229 (75.3%) | 123,307 (66.8%) |
| Positive | 207,831 (61.8%) | 898 (43.3%) | 7,086 (51.3%) | 5,721 (54.9%) | 431,184 (56.9%) | 1,843 (36.8%) | 16,394 (44.6%) | 11,804 (47.2%) | 2,963,496 (59.6%) | 10,432 (39.1%) | 118,016 (45.6%) | 92,087 (49.0%) | 1,016,748 (50.2%) | 2,043 (24.7%) | 61,354 (33.2%) |
| **First vaccine type** | | | | | | | | | | | | | | | |
| Ad26 | 0 (NA%) | 0 (NA%) | 0 (NA%) | 0 (NA%) | 154,639 (20.4%) | 723 (14.4%) | 8,419 (22.9%) | 6,378 (25.5%) | 117,208 (2.4%) | 559 (2.1%) | 7,865 (3.0%) | 5,891 (3.1%) | 107 (0.0%) | 1 (0.0%) | 15 (0.0%) |
| AZ | 0 (NA%) | 0 (NA%) | 0 (NA%) | 0 (NA%) | 188,571 (24.9%) | 1,144 (22.8%) | 8,812 (24.0%) | 6,227 (24.9%) | 2,144,213 (43.2%) | 10,206 (38.2%) | 103,140 (39.8%) | 81,943 (43.6%) | 925,574 (45.7%) | 3,446 (41.7%) | 80,971 (43.8%) |
| BNT162b2 | 0 (NA%) | 0 (NA%) | 0 (NA%) | 0 (NA%) | 266,607 (35.2%) | 1,774 (35.4%) | 12,568 (34.2%) | 8,083 (32.3%) | 1,541,424 (31.0%) | 8,077 (30.3%) | 86,843 (33.5%) | 57,792 (30.8%) | 154,979 (7.7%) | 395 (4.8%) | 16,609 (9.0%) |
| CV | 0 (NA%) | 0 (NA%) | 0 (NA%) | 0 (NA%) | 148,225 (19.6%) | 1,367 (27.3%) | 6,972 (19.0%) | 4,300 (17.2%) | 1,166,235 (23.5%) | 7,857 (29.4%) | 61,025 (23.6%) | 42,136 (22.4%) | 944,691 (46.6%) | 4,430 (53.6%) | 87,066 (47.1%) |
| Unknown | 336,468 | 2,074 | 13,810 | 10,418 | 0 | 0 | 0 | 0 | 0 | 0 | 0 | 0 | 0 | 0 | 0 |
| **Second vaccine type** | | | | | | | | | | | | | | | |
| Ad26 | 0 (NA%) | 0 (NA%) | 0 (NA%) | 0 (NA%) | 0 (NA%) | 0 (NA%) | 0 (NA%) | 0 (NA%) | 94,477 (1.9%) | 444 (1.7%) | 6,560 (2.5%) | 4,960 (2.6%) | 139 (0.0%) | 0 (0.0%) | 12 (0.0%) |
| AZ | 0 (NA%) | 0 (NA%) | 0 (NA%) | 0 (NA%) | 0 (NA%) | 0 (NA%) | 0 (NA%) | 0 (NA%) | 2,005,276 (40.4%) | 9,424 (35.3%) | 96,558 (37.3%) | 76,711 (40.9%) | 914,114 (45.1%) | 3,415 (41.3%) | 79,689 (43.2%) |
| BNT162b2 | 0 (NA%) | 0 (NA%) | 0 (NA%) | 0 (NA%) | 0 (NA%) | 0 (NA%) | 0 (NA%) | 0 (NA%) | 1,736,438 (34.9%) | 9,192 (34.4%) | 95,979 (37.1%) | 65,623 (35.0%) | 166,548 (8.2%) | 431 (5.2%) | 17,859 (9.7%) |
| CV | 0 (NA%) | 0 (NA%) | 0 (NA%) | 0 (NA%) | 0 (NA%) | 0 (NA%) | 0 (NA%) | 0 (NA%) | 1,132,889 (22.8%) | 7,639 (28.6%) | 59,776 (23.1%) | 40,468 (21.6%) | 944,550 (46.6%) | 4,426 (53.5%) | 87,101 (47.2%) |
| Unknown | 336,468 | 2,074 | 13,810 | 10,418 | 758,042 | 5,008 | 36,771 | 24,988 | 0 | 0 | 0 | 0 | 0 | 0 | 0 |
| **Third vaccine type** | | | | | | | | | | | | | | | |
| Ad26 | 0 (NA%) | 0 (NA%) | 0 (NA%) | 0 (NA%) | 0 (NA%) | 0 (NA%) | 0 (NA%) | 0 (NA%) | 0 (NA%) | 0 (NA%) | 0 (NA%) | 0 (NA%) | 19,903 (1.0%) | 80 (1.0%) | 2,276 (1.2%) |
| AZ | 0 (NA%) | 0 (NA%) | 0 (NA%) | 0 (NA%) | 0 (NA%) | 0 (NA%) | 0 (NA%) | 0 (NA%) | 0 (NA%) | 0 (NA%) | 0 (NA%) | 0 (NA%) | 91,285 (4.5%) | 340 (4.1%) | 8,577 (4.6%) |
| BNT162b2 | 0 (NA%) | 0 (NA%) | 0 (NA%) | 0 (NA%) | 0 (NA%) | 0 (NA%) | 0 (NA%) | 0 (NA%) | 0 (NA%) | 0 (NA%) | 0 (NA%) | 0 (NA%) | 1,804,225 (89.1%) | 7,537 (91.1%) | 165,949 (89.9%) |
| CV | 0 (NA%) | 0 (NA%) | 0 (NA%) | 0 (NA%) | 0 (NA%) | 0 (NA%) | 0 (NA%) | 0 (NA%) | 0 (NA%) | 0 (NA%) | 0 (NA%) | 0 (NA%) | 109,938 (5.4%) | 315 (3.8%) | 7,859 (4.3%) |
| Unknown | 336,468 | 2,074 | 13,810 | 10,418 | 758,042 | 5,008 | 36,771 | 24,988 | 4,969,080 | 26,699 | 258,873 | 187,762 | 0 | 0 | 0 |
| **Hospitalization** | 15,314 (4.6%) | 31 (1.5%) | 136 (1.0%) | 154 (1.5%) | 10,358 (1.4%) | 46 (0.9%) | 199 (0.5%) | 157 (0.6%) | 61,263 (1.2%) | 153 (0.6%) | 1,117 (0.4%) | 1,054 (0.6%) | 55,337 (2.7%) | 163 (2.0%) | 2,014 (1.1%) |
| **Death** | 5,624 (1.7%) | 8 (0.4%) | 32 (0.2%) | 32 (0.3%) | 3,042 (0.4%) | 11 (0.2%) | 46 (0.1%) | 36 (0.1%) | 18,151 (0.4%) | 31 (0.1%) | 228 (0.1%) | 198 (0.1%) | 16,870 (0.8%) | 33 (0.4%) | 337 (0.2%) |
| **Severe outcome** | 16,292 (4.8%) | 32 (1.5%) | 141 (1.0%) | 162 (1.6%) | 10,960 (1.4%) | 48 (1.0%) | 211 (0.6%) | 162 (0.6%) | 64,518 (1.3%) | 165 (0.6%) | 1,185 (0.5%) | 1,105 (0.6%) | 57,961 (2.9%) | 168 (2.0%) | 2,095 (1.1%) |

Ad26: Ad26.COV2.S vaccine; AZ: ChAdOx1 nCoV-19 vaccine; BNT162b2: BNT162b2 vaccine; CV: CoronaVac vaccine; RT-PCR: reverse transcription polymerase chain reaction.

**S5 Table:** Test-negative design analysis demonstrating odds ratios (OR) and associated 95% confidence intervals (CI) for risk of symptomatic infection and severe disease outcomes by history of previous infection and vaccination status in Brazil during the pre-Omicron period.

|  | **Symptomatic COVID-19** | | | **Severe COVID-19** | | |
| --- | --- | --- | --- | --- | --- | --- |
|  | **OR** | **LCL** | **UCL** | **OR** | **LCL** | **UCL** |
| Unvaccinated and no natural infection (Reference) | 1.00 | - | - | 1.00 | - | - |
| Only natural infection <6 months previously | 0.19 | 0.18 | 0.19 | 0.10 | 0.09 | 0.11 |
| Only natural infection 6 - 11 months previously | 0.19 | 0.19 | 0.19 | 0.10 | 0.08 | 00.12 |
| Only natural infection ≥12 months previously | 0.23 | 0.22 | 0.24 | 0.08 | 0.08 | 0.09 |
| 1 vaccination only | 0.68 | 0.68 | 0.69 | 0.37 | 0.37 | 0.38 |
| 1 vaccination + previous infection <6 months previously | 0.10 | 0.10 | 0.10 | 0.03 | 0.02 | 0.03 |
| 1 vaccination + previous infection 6-11 months previously | 0.10 | 0.10 | 0.10 | 0.03 | 0.03 | 0.03 |
| 1 vaccination + previous infection ≥12 months previously | 0.12 | 0.12 | 0.13 | 0.04 | 0.03 | 0.05 |
| 2 vaccinations only | 0.52 | 0.52 | 0.53 | 0.24 | 0.24 | 0.24 |
| 2 vaccinations + previous infection <6 months previously | 0.10 | 0.10 | 0.11 | 0.02 | 0.02 | 0.03 |
| 2 vaccinations + previous infection 6-11 months previously | 0.12 | 0.11 | 0.12 | 0.02 | 0.02 | 0.02 |
| 2 vaccinations + previous infection ≥12 months previously | 0.16 | 0.15 | 0.16 | 0.03 | 0.02 | 0.03 |
| 3 vaccinations only | 0.19 | 0.19 | 0.20 | 0.06 | 0.05 | 0.07 |
| 3 vaccinations + previous infection <6 months previously | 0.08 | 0.06 | 0.10 | 0.03 | 0.01 | 0.05 |
| 3 vaccinations + previous infection >6 months previously | 0.12 | 0.10 | 0.12 | 0.02 | 0.02 | 0.03 |

**S6 Table:** Characteristics of the study population for the nested case control design analysis for Brazil and Scotland.

|  | | **Brazil** | | **Scotland** | |
| --- | --- | --- | --- | --- | --- |
|  |  | **Cases**  (n=8,229) | **Controls**  (n=657,488) | **Cases**  (n=246) | **Controls**  (n= 826,726) |
| **Sex** | Female | 4,073 (51%) | 325,379 (51%) | 149 (61%) | 433,823 (52%) |
| **Age** | ≥60 years | 4,042 (49%) | 322,728 (49%) | 135 (55%) | 113,545 (14%) |
| **Number of medical comorbidities** | 0 | 2,543 (31%) | 474,198 (72%) | 57 (23%) | 523,680 (63%) |
|  | 1 | 2,396 (29%) | 119,708 (18%) | 52 (21%) | 216,300 (26%) |
|  | 2 | 2,100 (26%) | 52,208 (8.0%) | 45 (18%) | 57,928 (7.0%) |
|  | 3+ | 1,190 (14%) | 11,074 (1.7%) | 92 (37%) | 28,818 (3.5%) |
