# Supplementary material for: Sustained natural immunity following SARS-CoV-2 infection against severe COVID-19 outcomes and symptomatic reinfection: analyses of national data for Brazil and Scotland
Source: BMJ Open. 2025 Jul 16;15(7):e104057. doi: 10.1136/bmjopen-2025-104057 (PMC12273164; doi:10.1136/bmjopen-2025-104057)
Supplement: Supplementary file 2 [file bmjopen-2025-104057.reviewer_comments.pdf]

PEER REVIEW HISTORY

BMJ Open publishes all reviews undertaken for accepted manuscripts. Reviewers are asked to complete a checklist review form and are provided with free text boxes to elaborate on their assessment. These free text comments are reproduced below.

ARTICLE DETAILS

Title (Provisional)

Sustained natural immunity following SARS-CoV-2 infection against severe COVID-19 outcomes and symptomatic reinfection: Analyses of national data for Brazil and Scotland

Authors

Haider, Fasih; Cerqueira-Silva, Thiago; Hainey, Kirsten J.; Millington, Tristan; Shah, Syed Ahmar; de Araujo Oliveira, Vincius; Pearce, Neil; Barreto, Mauricio L; Boaventura, Viviane Sampaio; Katikireddi, Srinivasa Vittal; Robertson, Chris; Barral-Netto, Manoel; Sheikh, Aziz

---

VERSION 1 - REVIEW

Reviewer **1**

Name **Abu-Raddad, Laith**

Affiliation **Weill Cornell Medical College in Qatar, Infectious Disease Epidemiology Group**

Date **11-May-2025**

COI **None**

Thank you for considering the suggestions and comments and revising the manuscript.

Reviewer **2**

Name **Valentiner-Branth, Palle**

Affiliation **Statens Serum Institut**

Date **26-May-2025**

COI **None**

The reviewer comments have been satisfactorily addressed
